# The risk of mortality and severe illness in patients infected with the omicron variant relative to delta variant of SARS-CoV-2: a systematic review and meta-analysis

**DOI:** 10.1007/s11845-022-03266-6

**Published:** 2023-02-09

**Authors:** Chia Siang Kow, Dinesh Sangarran Ramachandram, Syed Shahzad Hasan

**Affiliations:** 1 School of Pharmacy, International Medical University, Kuala Lumpur, Malaysia; 2 School of Pharmacy, Monash University Malaysia, Bandar Sunway, Selangor, Malaysia; 3 School of Applied Sciences, University of Huddersfield, Huddersfield, UK; 4 School of Biomedical Sciences & Pharmacy, University of Newcastle, Callaghan, Australia

**Keywords:** Coronavirus, Delta, Omicron, Variant

## Abstract

We summarized through systematic review and meta-analysis of observational studies the risk of mortality as well as severe illness of COVID-19 caused by omicron variant relative to delta variant of SARS-CoV-2. A total of twelve studies were included. Our results showed significantly reduced odds of mortality (pooled OR = 0.33; 95% CI: 0.16–0.67) and significantly reduced odds of severe illness (pooled OR = 0.24; 95% CI: 0.21–0.28) in patients infected with the omicron variant of SARS-CoV-2 relative to their counterparts infected with the delta variant. Findings of lower disease severity following infection with the omicron variant of SARS-CoV-2 than the delta variant are encouraging during the ongoing transition from the pandemic phase into the endemic phase of COVID-19.

## Introduction

The omicron variant (B.1.1.529) of severe acute respiratory syndrome coronavirus 2 (SARS-CoV-2), which is the virus that causes coronavirus disease 2019 (COVID-19), was first reported on November 24, 2021, to the World Health Organization (WHO) from South Africa [1, 2]. Important questions remain as to the clinical impact of the omicron variant [3]. The Delta variant of SARS-CoV-2 was reported in a systematic review and meta-analysis [4] to cause more severe illness than previous variants. Therefore, concerns arise regarding the severity of infection caused by the omicron variant of SARS-CoV-2, since the emergence of a new variant of concern, is more likely to lead to increased pathogenicity, based on previous experiences [5]. In this paper, we aimed to summarize through systematic review and meta-analysis of observational studies the overall risk of mortality as well as severe illness of COVID-19 caused by the omicron variant relative to the delta variant of SARS-CoV-2.

## Methods

### Literature screening

We performed a systematic literature search with no language restriction in electronic databases, including PubMed, Web of Science, Scopus, Google Scholar, and preprint servers (medRxiv, Research Square, SSRN), to identify relevant studies involving only human subjects from inception until June 07, 2022 [6]. The search strategy in the electronic databases was built based on the following keywords and their MeSH terms (if applicable): “COVID-19,” “SARS-CoV-2,” “b.1.1.529,” “omicron,” “ba.1,” and “ba.2.” In addition, we performed manual searches of the cited references of relevant articles to retrieve additional studies.

### Study selection

Two investigators (CSK and SSH) independently performed the literature screening to identify eligible studies. Studies were eligible for inclusion in the systematic review and meta-analysis if they were observational studies comparing the risk of COVID-19-associated mortality or the risk of COVID-19-associated severe illness, between patients with COVID-19 infected with the omicron variant of SARS-CoV-2 and those infected with the delta variant, and reported the adjusted estimates of odds ratio, hazard ratio, or relative risk (RR), with corresponding 95% confidence intervals. We excluded observational studies that reported non-adjusted estimates, as well as comments, case reports, conference papers, animal experiments, letters, and review articles which reported no original data. In addition, studies that did not identify the variants of SARS-CoV-2 via sequencing, genotyping, or S-gene positivity were also excluded.

### Outcomes

The outcomes of interest were COVID-19-associated fatal illness and COVID-19-associated severe illness, which included admission to the intensive care unit, the requirement of ventilation, and/or as defined by the investigators.

### Data extraction

Two investigators (CSK and DSR) extracted the main characteristics of each study. Disagreements concerning data extraction were resolved by discussion between the two investigators.

### Risk of bias assessment

Newcastle–Ottawa Scale [7] was used for critical appraisal of the methodological quality of included observational studies, wherein the included studies could be categorized as low, moderate, and high quality with the scores of 0–5, 6–7, and 8–9, respectively [8]. Two investigators (CSK and DSR) independently assessed the quality of each study. Any conflicts in the assessment were solved through discussion between the two investigators.

### Statistical analysis

Meta-analysis with the random-effects model was used to estimate the pooled odds/hazard ratio of mortality and the pooled odds/hazard ratio of severe illness in patients with COVID-19 infected with SARS-CoV-2 of omicron variant relative to their counterparts infected with delta variants, at 95% confidence intervals. Heterogeneity was quantified using the *I*^2^ statistics and the *χ*^2^ test, with statistically significant heterogeneity predetermined at *I*^2^ of > 50% and *P*-value of < 0.10, respectively. All statistical analyses were performed using Meta XL, version 5.3 (EpiGear International, Queensland, Australia).

## Results

### Literature search

Our systematic literature search yielded 5,759 potential studies, of which 2,017 were unique (records retrieved after removing duplications). After the initial screening of titles and abstracts, 14 articles were retained for full-text review. Upon screening against eligibility criteria, twelve observational studies [3, 9–19] were ultimately included. Table 1 shows the characteristics of the included studies [3, 9–19] in detail.

**Table 1:** Characteristics of included studies

**Study (year)**	**Study design**	**Country**	**Number of patients/cases**	**Age (median/mean)**	**Proportion of patients/cases who had been vaccinated (%)**	**Proportion of patients/cases with severe illness**^**a**^		**Adjusted estimate of severe illness**	**Proportion of patients/cases with fatal illness**		**Adjusted estimate of fatal illness**	**Adjusted covariates**	**NOS**
						**Omicron variant (*n*/*N*; %)**	**Delta variant (*n/N*; %)**		**Omicron variant (*n*/*N*; %)**	**Delta variant (*n*/*N*; %)**			
**Wolter et al. **[9]	Retrospective database review	South Africa	1037	N/A	Total = 55.3	57/244; 23.4	496/793; 62.5	OR = 0.30 (0.20–0.50)	-	-	-	Age, sex, comorbidity, province, type of health-care sector, number of days between specimen collection and hospital admission, known previous SARS-CoV-2 infection, SARS-CoV-2 vaccination status	8
**Peralta-Santos et al. **[10]	Retrospective database review	Portugal	15,978	Omicron variant = 37.1 Delta variant = 43.4	Omicron variant = 88.1 Delta variant = 89.0	-	-	-	0/6581; 0	26/9397; 0.3	OR = 0.14 (0.01–1.12)	Age, sex, known previous SARS-CoV-2 infection, SARS-CoV-2 vaccination status	8
**Auvigne et al. **[11]	Retrospective database review	France	184,364	N/A	Omicron variant = 88.7 Delta variant = 67.2	N/A	N/A	HR = 0.12 (0.08–0.18)	-	-	-	Age, sex, comorbidity, SARS-CoV-2 vaccination status, region of residence	8
**Ward et al. **[12]	Retrospective database review	UK	1,035,163	N/A	Total = 89.1	-	-	-	N/A	N/A	HR = 0.33 (0.24–0.45)	Age, sex, SARS-CoV-2 vaccination status, known previous SARS-CoV-2 infection, calendar time, ethnicity, Index of Multiple Deprivation rank, household deprivation, university degree, keyworker status, country of birth, main language, region, disability, health risk factors	8
**Šmíd et al. **[13]	Retrospective database review	Czech Republic	312,152	N/A	Total = 55.8	N/A	N/A	OR = 0.24 (0.21–0.28)	-	-	-	Age, sex, SARS-CoV-2 vaccination status	8
**Stålcrantz et al. **[14]	Retrospective database review	Norway	1075	Omicron variant = 55 Delta variant = 59	Omicron variant = 75.8 Delta variant = 39.8	31/409; 7.6	165/666; 24.8	HR = 0.52 (0.34–0.80)	15/379; 4.0	63/631; 10.0	HR = 0.44 (0.24–0.79)	Age, sex, SARS-CoV-2 vaccination status, country of birth, underlying risk factors, regional health authority	6
**Harrigan et al. **[15]	Retrospective database review	Canada	13,128	Omicron variant = 35.0 Delta variant = 34.4	Omicron variant = 90.5 Delta variant = 53.0	15/7729; 0.2	115/5399; 2.1	HR = 0.27 (0.19–0.38)	-	-	-	Age, sex, geography, Elixhauser index, neighbourhood income quintile	8
**Nyberg et al. **[16]	Retrospective database review	UK	1,516,702	N/A	Omicron variant = 82.0 Delta variant = 58.1	-	-	-	1225/106785; 0.11	1205/448843; 0.27	HR = 0.31 (0.26–0.37)	Age, sex, index of multiple deprivation, SARS-CoV-2 vaccination status, known previous SARS-CoV-2 infection	8
**Gunadi et al. **[17]	Retrospective database review	Indonesia	352	Omicron variant = 39.1 Delta variant = 36.5	N/A	-	-	-	10/139; 7.2	19/213; 8.9	OR = 1.15 (0.45–2.98)	Age, sex, comorbidity, smoking status	8
**Sievers et al. **[18]	Retrospective database review	Germany	59,681	N/A	Omicron variant = 66.4 Delta variant = 41.8	BA.1: 43/15734; 0.3 BA.2: 19/6846; 0.3	403/24416; 1.7	BA.1 vs Delta variant: OR = 0.20 (0.12–0.32) BA.2 vs Delta variant: OR = 0.17 (0.07–0.39)	BA.1: 96/20818; 0.5 BA.2: 26/7143; 0.4	545/31720; 1.7	BA.1 vs Delta variant: OR = 0.38 (0.25–0.58) BA.2 vs Delta variant: OR = 0.16 (0.08–0.30)	Age, sex, SARS-CoV-2 vaccination status, federal state of notifying health authority, week of notification	8
**Lewnard et al. **[19]	Retrospective database review	USA	245,993	N/A	Omicron variant = 70.6 Delta variant = 57.9	N/A	N/A	HR = 0.48 (0.29–0.81)	N/A	N/A	HR = 0.21 (0.10–0.44)	Age, sex, SARS-CoV-2 vaccination status, known previous SARS-CoV-2 infection, race/ethnicity, community median income, smoking status, body mass index, prior year outpatient visits, prior year emergency department visits, prior year inpatient admissions, Charlson comorbidity index,	8
**Fall et al. **[3]	Retrospective cohort study	USA	2027	Omicron variant = 32.0 Delta variant = 35.0	Omicron variant = 17.6 Delta variant = 54.9	10/1119; 0.9	41/908; 4.5	OR = 0.39 (0.17–0.89)	3/1119; 0.3	21/908; 2.3	OR = 0.22 (0.05–0.91)	Age, sex, race/ethnicity, comorbidity	8

*NOS* Newcastle–Ottawa Scale, *OR* odds ratio, *HR* hazard ratio

^a^The definition of severe illness varied across the included studies; in the study reported by Šmíd et al. [13], Stålcrantz et al. [14], Harrigan et al. [15], Sievers et al. [18], Lewnard et al. [19], and Fall et al. [3], severe illness was defined as admission into intensive care unit; in the study reported by Auvigne et al. [11], severe illness was defined as admission into intensive care unit and/or death; and in the study reported by Wolter et al. [9], severe illness was defined as admission into intensive care unit, requirement for supplemental oxygen, requirement for ventilation, requirement for extracorporeal membrane oxygenation, development of acute respiratory distress syndrome, and/or death

### Study characteristics

Across the twelve included studies [3, 9–19], all but one are retrospective database reviews [9–19]; the remaining one study [3] is a retrospective cohort study. The included studies [9–19] were performed in nine countries, including South Africa [9], Portugal [10], France [11], the UK (*n* = 2) [12, 16], Czech Republic [13], Norway [14], Canada [15], Indonesia [17], Germany [18], and the USA (*n* = 2) [3, 19]. The average age of the analyzed patients across the included studies ranged from 32.0 to 59.0. Age and sex were the most commonly adjusted covariates, followed by SARS-CoV-2 vaccination status.

Eight of the included studies [3, 9, 11, 13–15, 18, 19] reported adjusted estimates for severe illness between patients infected with the omicron variant of SARS-CoV-2 and those infected with the delta variant. The definition of severe illness varied across the included studies (Table 1). On the other hand, eight of the included studies [3, 10, 12, 14, 16–19] reported adjusted estimates for mortality between patients infected with the omicron variant of SARS-CoV-2 and those infected with the delta variant.

### Study quality

The included studies were assessed for methodological quality with Newcastle–Ottawa Scale. All except one of the included studies [3, 9–13, 15–19] were deemed high quality with a Newcastle–Ottawa Scale of 8 (Table 1); the remaining study [14] was of moderate quality with a Newcastle–Ottawa Scale of 6.

### Risk of mortality (fatal illness)

**Fig. 1:**
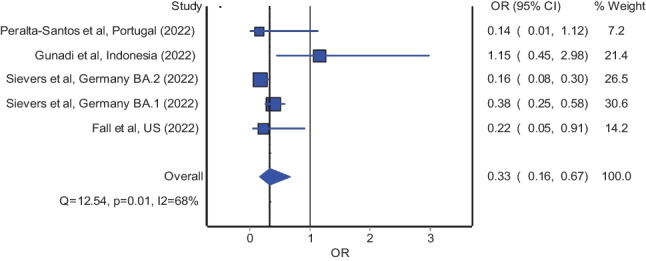
Pooled odds ratio of mortality in patients infected with the omicron variant relative to the delta variant of SARS-CoV-2

The meta-analysis of four studies [3, 10, 17, 18] which reported adjusted estimates in odds ratio revealed significantly reduced odds of mortality in patients infected with the omicron variant of SARS-CoV-2 relative to their counterparts infected with the delta variant; the estimated effect indicates reduced mortality (Fig. 1; pooled odds ratio = 0.33; 95% confidence interval: 0.16 to 0.67) and is with adequate evidence to reject the model hypothesis of “no significant difference,” at the current sample size. Likewise, the meta-analysis of four studies [12, 14, 16, 19] which reported adjusted estimates in hazard ratio also demonstrated significantly reduced mortality hazards in patients infected with the omicron variant of SARS-CoV-2 relative to the delta variant (pooled hazard ratio = 0.32; 95% confidence interval: 0.28 to 0.37).

### Risk of severe illness

**Fig. 2:**
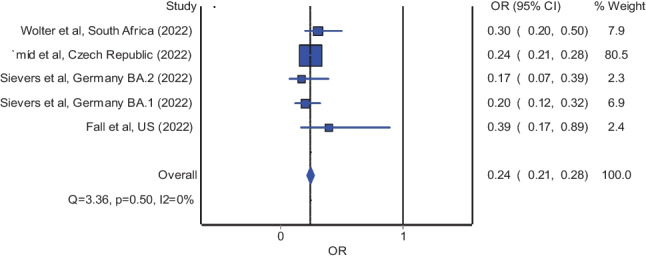
Pooled odds ratio of severe illness in patients infected with the omicron variant relative to the delta variant of SARS-CoV-2

The meta-analysis of four studies [3, 9, 13, 18] which reported adjusted estimates in odds ratio revealed significantly reduced odds of severe illness in patients infected with the omicron variant of SARS-CoV-2 relative to their counterparts infected with the delta variant; the estimated effect indicates a reduced risk of severe illness (Fig. 2; pooled odds ratio = 0.24; 95% confidence interval: 0.21 to 0.28) and is with adequate evidence to reject the model hypothesis of “no significant difference,” at the current sample size. Likewise, the meta-analysis of four studies [11, 14, 15, 19] which reported adjusted estimates in hazard ratio also demonstrated significantly reduced hazard of severe illness in patients infected with the omicron variant of SARS-CoV-2 relative to the delta variant (pooled hazard ratio = 0.26; 95% confidence interval: 0.12 to 0.56).

## Discussion

Our findings suggest that despite reports of increased transmissibility, the omicron variant does not lead to increased pathogenicity compared to the delta variant of the SARS-CoV-2, in the background of reduced vaccine effectiveness [20]. It is still unclear as to the reason for reduced severity of illness following infection with the omicron variant of SARS-CoV-2 than the delta variant, since it has not been inevitable that viral evolution leads to a lower severity. The risk of severe illness had been reported to increase significantly in patients infected with the delta variant of SARS-CoV-2 compared with the previous circulating variants [4]. Moreover, the risk of severe illness was also significantly increased with infection of the alpha variant of SARS-CoV-2 compared with the previously circulating lineages [5]. Nevertheless, the lower replication ability of the omicron variant in human lungs, as demonstrated in the ex vivo and in vivo models, is compatible with the reduced severity of illness as observed in our analyses [21, 22].

Nonetheless, there are concerns with the emergence of new omicron subvariants (especially BA.2), which may lead to increased virulence. Our systematic review identified only one study [18] which observed a similar reduction in mortality risk and severe illness risk with either the BA.1 or BA.2 omicron subvariant compared to the delta variant, which suggests no difference in pathogenicity between the two subvariants. Yet, due to a lack of available studies in the literature thus far, there is a fundamental need to perform more investigations on the relative virulence of omicron subvariants, especially the BA.1.1 subvariant. The characteristics of the illness, such as viral replication in the respiratory tract and development of interstitial pneumonia with infection caused by the BA.1.1 subvariant, were similar to the infection caused by the delta variant in Syrian hamsters [23].

Evidence of lower disease severity following infection with the omicron variant of SARS-CoV-2 than the delta variant is encouraging during the ongoing transition from the pandemic phase into the endemic phase of COVID-19. Nevertheless, genomic surveillance of SARS-CoV-2 should remain at the forefront of the global COVID-19 response to allow timely detection and characterization of new SARS-CoV-2 lineages, especially when it is not guaranteed that the emergence of new variants has a similarly reduced severity of illness.

## Data Availability

The data presented in this study are available in the manuscript.
